# Growth differentiation factor 15: a prognostic marker for recurrence in colorectal cancer

**DOI:** 10.1038/bjc.2011.112

**Published:** 2011-04-05

**Authors:** U Wallin, B Glimelius, K Jirström, S Darmanis, R Y Nong, F Pontén, C Johansson, L Påhlman, H Birgisson

**Affiliations:** 1 Division of Colon and Rectal Surgery, Department of Surgery, University of Minnesota, 2800 Medical Building, 2800 Chicago Avenue South, Suite 300, Minneapolis, MN, USA; 2 Department of Surgical Sciences, Colorectal Surgery, Uppsala University, Uppsala, Sweden; 3 Department of Oncology, Radiology and Clinical Immunology, Uppsala University, Uppsala, Sweden; 4 Department of Oncology and Pathology, Karolinska Institutet, Stockholm, Sweden; 5 Center for Molecular Pathology, Department of Laboratory Medicine, Skåne University Hospital, Lund University, Malmö, Sweden; 6 Department of Genetics and Pathology, Rudbeck Laboratory, Uppsala University, Uppsala, Sweden

**Keywords:** GDF15, colorectal cancer, prognostic markers, MIC-1

## Abstract

**Background:**

Growth differentiation factor 15 (GDF15) belongs to the transforming growth factor beta superfamily and has been associated with activation of the p53 pathway in human cancer. The aim of this study was to assess the prognostic value of GDF15 in patients with colorectal cancer (CRC).

**Methods:**

Immunohistochemistry and tissue microarrays were used to analyse GDF15 protein expression in 320 patients with CRC. In a subgroup of 60 patients, the level of GDF15 protein in plasma was also measured using a solid-phase proximity ligation assay.

**Results:**

Patients with CRC with moderate to high intensity of GDF15 immunostaining had a higher recurrence rate compared with patients with no or low intensity in all stages (stages I–III) (HR, 3.9; 95% CI, 1.16–13.15) and in stage III (HR, 10.32; 95% CI, 1.15–92.51). Patients with high plasma levels of GDF15 had statistically shorter time to recurrence (*P*=0.041) and reduced overall survival (*P*=0.002).

**Conclusion:**

Growth differentiation factor 15 serves as a negative prognostic marker in CRC. High expression of GDF15 in tumour tissue and high plasma levels correlate with an increased risk of recurrence and reduced overall survival.

Colorectal cancer (CRC) is the third most common cancer in the Western world. Globally, the age-standardised incidence rate for CRC is 20.1 per 100 000 males and 14.6 per 100 000 females (Parkin *et al*, 2005). Surgery is the most important treatment, but a substantial number of patients will get a recurrence, a risk being stage-dependent. The use of adjuvant treatment with 5-fluorouracil (5-FU) and folinic acid, after curative surgical resection for CRC, reduces the relative risk of recurrence by 30–35% and the addition of oxaliplatin reduces it even further (about 15–20%) (Ragnhammar *et al*, 2001; Andre *et al*, 2004). The relative risk reduction appears to be relatively independent of stage (Gill *et al*, 2004). In patients with node-positive disease (stage III), the gains translate into meaningful improvements in overall survival, but in patients with node-negative disease (stage II), the survival gain is only in the order of a few percent (Gray *et al*, 2007). It is not clear which all patients in stages II and III will benefit from adjuvant treatment and which in all patients, chemotherapeutic drug/s will be most effective. Consequently, there is a need for individualised therapy in patients curatively operated for CRC stages II and III.

Several prognostic and predictive markers have been identified, although very few of them are currently used in clinical practice. Microsatellite instability (MSI) is one prognostic factor in use for recurrence and overall survival in patients with CRC stages II and III (Watanabe *et al*, 2001; Popat *et al.*, 2005). Other identified prognostic markers in primary disease are thymidylate synthase and loss of heterozygosity of 18q, but these are currently not used in clinical practice. No predictive marker for response to adjuvant therapy has yet reached clinical use, although some studies show that MSI tumours do not adequately respond to 5-FU (Hemminki *et al*, 2000; Elsaleh *et al*, 2001; Jover *et al*, 2006; Ohrling *et al*, 2010). Mutations in *KRAS* are clinically used as a predictor for poor response to treatment with EGFR-directed antibodies in patients with metastatic CRC (Amado *et al*, 2008; Karapetis *et al*, 2008).

Growth differentiation factor 15 (GDF15) belongs to the transforming growth factor beta superfamily and has a role in regulating inflammatory and apoptotic pathways in injured tissues and during disease processes. Under normal conditions, the placenta is the only tissue that expresses GDF15 in significant amounts; however, small amounts of GDF15 mRNA can be detected in a few other tissues, including the kidneys, pancreas, prostate and colon (Fairlie *et al*, 1999). Growth differentiation factor 15, also known as PTGF-*β*, TGF-PL or MIC-1, has been hypothesised to serve as a secreted biomarker for activation of the p53 pathway in human cancer (Seetoo *et al*, 2003; Koopmann *et al*, 2004; Weber *et al*, 2005; de Wit *et al*, 2005; Brown *et al*, 2006), and in CRC MIC-1, serum levels and genotype were associated with decreased overall survival and extent of disease (Brown *et al*, 2003; Xue *et al*, 2010). Immunohistochemical analysis of GDF15 expression in CRC has also been associated with lymph node metastasis (Xue *et al*, 2010).

Our aim was to further validate the prognostic value of immunohistochemical GDF15 expression and GDF15 levels in plasma in a defined cohort of patients operated for CRC.

## Patients and methods

### Patients

A population-based cohort of 320 patients with CRC, treated between August 2000 and December 2003 at the Central district Hospital in Västerås, were prospectively included to participate in the study after giving informed consent. The median follow-up time was 6 years (8–10) in surviving patients. Information about tumour size, grade, stage and location, neurovascular tumour infiltration, lymph node involvement and mucinous component were obtained from the pathology records, and had, thus, been assessed by different pathologists. Selection of tumour areas used for tissue microarrays (TMAs) was made by one single pathologist (KJ). Information about cancer recurrence, cause of death and the use of neo-adjuvant/adjuvant treatment was obtained from surgical and oncology records and by matching with the Clinical Database for Colorectal cancer held at the Regional Oncologic Center in Uppsala/Örebro region.

### TMA construction

All cases were histopathologically re-evaluated on H&E stained tumour specimens by one pathologist (KJ), and areas representative of normal mucosa, invasive tumour, lymph node metastases and, when present, adenomatous tissue were selected. A manual arraying device (MTA-1, Beecher Instruments Inc., Sun Prairie, WI, USA) was used for extraction of five 1.0-mm cores from each case; two from the invasive tumour, one from normal mucosa, one from adenomatous mucosa and one from a selected lymph node metastasis.

### Immunohistochemistry and annotation

**Figure 1:**
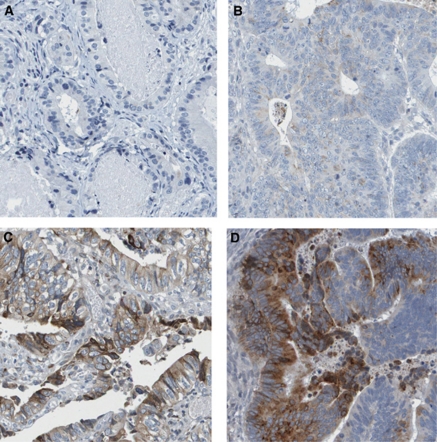
These images represent the four levels of the intensity of immunoreactivity, resulting from immunostaining with GDF15 antibody on primary colorectal cancer tissues. Negative (**A**), weak (**B**), moderate (**C**) and strong intensity staining (**D**). Images with immunostaining present had 25–75% fraction of positive cells.

Immunohistochemistry was performed on 4 *μ*m TMA sections using HPA011191 (Atlas Antibodies, Stockholm, Sweden) as primary antibody to detect GDF15. Automated immunohistochemistry (Autostainer 480, Lab Vision, Fremont, CA, USA) was performed as previously described (Paavilainen *et al*, 2010). Immunohistochemically stained TMA sections were scanned in high-resolution scanners (ScanScope T2, Aperio Technologies, Vista, CA, USA) and separated into individual spot images representing different cores in the TMAs. The annotation process included estimation of the intensity of immunoreactivity for GDF15 (negative (0), weak (1), moderate (2), or strong, (3) and fraction (%) of GDF15-positive cells (<1% (0), 1–24% (1), 25–75% (2), or >75% (3)) (Figure 1). Tumours with no (0) or low (1) intensity and no (0) or low (1) fraction of GDF15 expression were allocated to one group and tumours with moderate (2) or high (3) intensity and moderate (2) and high (3) fraction of GDF15 expression were allocated into a second group.

### Plasma GDF15 analyses

Pre-surgery EDTA plasma was analysed for GDF15 abundance. Patients initially included in the study were selected for GDF15 plasma analyses. The aim was to include 10 patients in each group after stratifying both for recurrence and stage. However, because of limitations in the number of cases in our cohort (i.e., recurrence in stage I) and limitations in availability of plasma samples, only nine patients with stage I (one recurrence), approximately 10 patients with and without recurrence from stages II and III, respectively, and 8 patients with stage IV were selected from the whole cohort. GDF15 was analysed by using SP-PLA (Darmanis *et al*, 2010). The protocol was modified to facilitate multiplex detection of various analytes, including GDF15, according to a method currently prepared for publication.

### Statistical analyses

For categorical data, the *χ*^2^-test was performed. All *P*-values were two sided, and statistical significance was set at *P*<0.05. Wilcoxon matched-paired sign test was used to compare GDF15 staining intensity and fraction between normal mucosa and tumour. Kruskal–Wallis ANOVA test was used to compare the median values of GDF15 plasma levels between different disease stages and the Mann–Whitney *U*-test was used to compare the median values of GDF15 plasma levels within each disease stage. The Kaplan–Meier method was used for survival analyses and comparison of strata performed by the log-rank test. Cox proportional multivariate analyses were performed to evaluate the statistical significance and independence of intensity and fraction of GDF15 expression and only variables with a *P*-value <0.10 in univariate analysis were included in the multivariate analyses. Patients without known metastases at the time of surgery and microscopically free resection margin (R0) were considered to be curatively operated. Overall survival was measured from the time of surgery to the time of death, irrespective of cause. Time to recurrence was calculated as time to any event related to the same cancer (Punt *et al*, 2007). Deaths from other cancers (*n*=4), non-cancer-related deaths (*n*=65), treatment-related deaths (*n*=6) and loss to follow-up (*n*=1) were censored. Second primary same cancers (*n*=4) and other primary cancers (*n*=22) were ignored. All observations were censored at the end of the study period (15 April 2010). Data were analysed using STATISTICA software (version 7.1, StatSoft Inc., Tulsa, OK, USA).

Ethical approval (no 00-001) was obtained from the Ethics Committee at Uppsala University, Uppsala, Sweden.

## Results

### Identification of GDF15 as a potential prognostic marker

The GDF15 antibody HPA011191 was generated within the Swedish Human Protein Atlas project (http://www.proteinatlas.org; http://www.proteinatlas.org/ ENSG00000130513/antibody) (Uhlen *et al*, 2005). The antibody was validated by protein epitope signature tag array analysis with high specificity and a supportive pattern was seen by immunohistochemistry, consistent with experimental and/or bioinformatic data. In addition, western blotting revealed a single band corresponding to the predicted size of 34 kDa. Protein profiling of GDF15 expression was performed using HPA011191 on TMAs containing normal tissues from 140 different individuals, cancer tissues from 216 patients and 47 cell lines (Ponten *et al*, 2008). GDF15 was selectively expressed with strong cytoplasmic immunostaining in trophoblastic cells from the placenta, and only a weak to moderate cytoplasmic expression in other normal cell types, including the colon and prostate. A differential expression pattern was found in cancer tissues representing several forms of cancer, within CRC ranging from negative tumours to tumours with strong GDF15 expression.

### Immunohistochemical analyses

The intensity of immunoreactivity (*P*<0.001) was higher in tumour tissue than in normal colon mucosa, but no difference in the fraction of positive cells was observed (*P*=0.19) (Table 1). In addition, no difference in fraction (*P*=0.36) or intensity (*P*=0.56) of GDF15 expression between adenoma and normal tissue was observed, however, the number of patients with adenomas in the cohort was low (*n*=9). Tumours expressing moderate or high intensity of GDF15 were less likely to have vascular invasion (*P*=0.036). No other histopathological or clinical parameters were correlated with the intensity or fraction of GDF15 in the tumour (Table 2).

### Survival analyses

**Figure 2:**
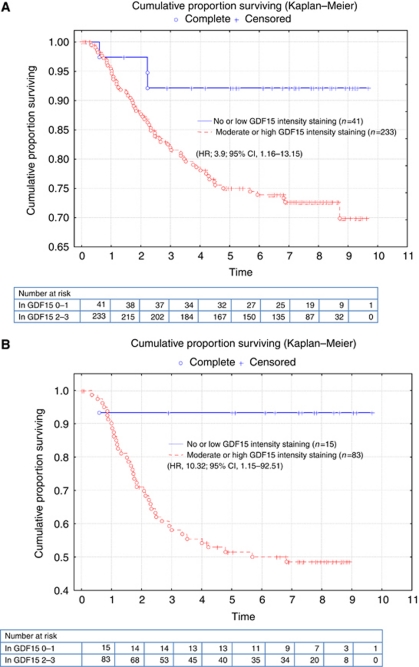
Time to recurrence according to the intensity of immunoreactivity of GDF15 in the primary invasive tumour tissue in patients curatively operated for CRC stages I–III (**A**) and III (**B**).

Patients curatively treated for CRC in stages I–III or in stage III with moderate to high intensity of immunoreactivity for GDF15 had a higher recurrence rate and shorter time to recurrence compared with patients with no or low intensity for GDF15 (Figures 2A and B). These differences were statistically significant in multivariate analysis (stages I–III, (HR, 3.9; 95% CI, 1.16–13.15); stage III (HR, 10.32; 95% CI, 1.15–92.51)) (Table 3). Neither the intensity nor the fraction of GDF15 had any influence on overall survival in the cohort.

In 42 out of 100 curatively treated patients with stage III disease, immunohistochemical staining for GDF15 could be assessed in lymph node metastases. The intensity of GDF15 staining was higher in the primary cancer compared with the lymph node metastases (*P*=0.035). Patients with moderate to high intensity of immunoreactivity of GDF15 in the lymph node metastases did not have any higher risk of recurrence compared with patients with no to low intensity of GDF15 (*P*=0.08). However, when the intensity of GDF15 in lymph node metastases was stratified to no to moderate *vs* high, high expression was associated with increased risk for recurrences in stage III CRC (HR 1.78; 95% CI, 1.59–38.03) in a multivariate Cox regression model including gender, heredity for CRC, N stage, CEA and neural invasion.

### Comparison with CEA

**Figure 3:**
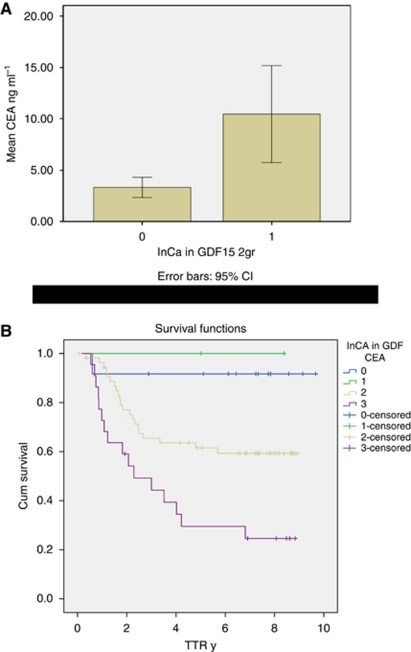
Curatively treated patients with colorectal cancer stages I–III (*n*=277). Box plot revealing higher CEA levels in those with increased expression of GDF15 defined as intensity of immunoreactivity (1) compared with low expression (0) (**A**). Time to recurrence in curatively treated patients with CRC and stage III divided into subgroups according to intensity of immunoreactivity for GDF15 and preoperative CEA levels. Group 0: intensity low (0–1) and CEA<6; Group 1 (*n*=12): intensity low (0–1) and CEA>6 (*n*=2); Group 2: intensity high (2–3) and CEA<6 (*n*=55); Group 3: intensity high (2–3) and CEA>6 (*n*=22) (**B**).

Low intensity of immunoreactivity in the primary tumour was associated with low levels of CEA (Figure 3A). When analysing patients with stage III disease, in which serum CEA was available for 91 of 100 patients, a decreased risk of recurrence was observed with low intensity of immunoreactivity for GDF 15 in the primary tumour independent of CEA level. In patients with high intensity of immunoreactivity for GDF15 and CEA>6 ng ml^−1^, an increased risk of recurrence was observed (HR 2.33; 95% CI, 1.15–4.42). This remained statistically significant in the multivariate analysis (including gender, heredity for CRC, N stage and neural invasion) (Figure 3B).

### Plasma analyses of GDF15

**Figure 4:**
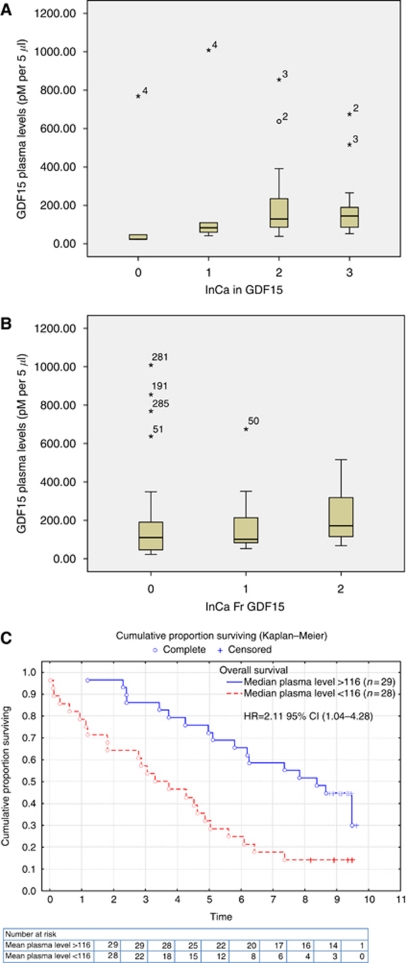
GDF15 plasma levels and intensity (**A**) and fraction (**B**) of immunohistochemistry expression of GDF15 in 57 patients with colorectal cancer stages I–IV. The boxes represents median and quantiles and bars minimum and maximum. Circles are outliers with values between 1.5 and 3 box lengths from the upper edge of the box and asterisks are extremes with values more than 3 box lengths from the upper edge of the box. Overall survival according to the median GDF15 plasma levels in patients operated for CRC stages I–IV (*n*=57). The multivariate Cox proportional analyses include gender, hereditary for CRC, N-substage and neural invasion (**C**).

Plasma levels of GDF15 were available for 57 patients, 28 without and 21 with recurrent disease and 8 with stage IV disease. A nonsignificant trend of elevated plasma levels of GDF15 was observed in tumours with increasing intensity (*P*=0.148) and fraction (*P*=0.326) of GDF15 expression as assessed by immunohistochemistry (Figures 4A and B). Patients with high plasma levels of GDF15 had a shorter time to recurrence (*P*=0.041) and a shorter overall survival (*P*=0.002) in the univariate analysis, and this remained significant for overall survival in multivariate analysis (HR 2.11; 95% CI, 1.04–4.28) (Figure 4C).

There was no significant difference in median GDF15 plasma levels between patients with or without recurrence when comparing all stages; however, in patients with stage III, a trend of a higher median plasma level of GDF15 was observed compared with patients in stage III without recurrence (*P*=0.072) (Table 4).

### Comparison between plasma levels of GDF15 and CEA

**Figure 5:**
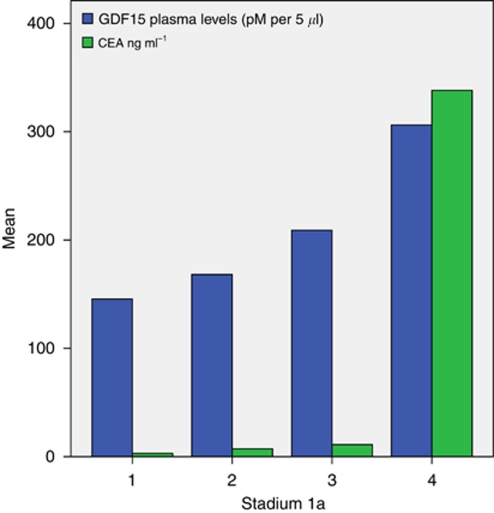
Mean preoperative plasma levels of GDF15 and CEA in patients with colorectal cancer stages I–IV (*n*=57).

The GDF15 plasma levels gradually increased with disease stage, whereas the CEA levels were low in stages I–III and markedly increased in stage IV (Figure 5). There was a weak correlation between the GDF15 plasma levels and CEA in the whole cohort (*P*<0.001; *r*=0.49), however, a stronger correlation was observed in stage IV (*P*=0.045; *r*=0.72).

## Discussion

In this study, we demonstrated a higher recurrence rate in patients curatively operated for CRC stages I–III with moderate or high intensity of GDF15 expression, compared with tumours with no or low intensity of GDF15 expression. This was also demonstrated separately for patients with stage III disease, but not for patients with stage II disease.

Our data are consistent with previous findings by Xue *et al* (2010) who investigated the expression of GDF15 in 69 CRC cases by immunohistochemistry. They demonstrated not only an association between upregulation of GDF15 and development of metastases but also, different from our study, an increased immunohistochemical GDF15 expression in stages III and IV compared with stages I and II. However, unlike our study, the study by Xue *et al* (2010) used a combined score to quantify the intensity and fraction of GDF15 immunostaining, thus, limiting further comparisons between the two studies.

Brown *et al* (2003) demonstrated an association between high GDF15 blood levels, presence of metastatic disease and an elevated risk of death. In our study, we observed that the risk of death was more than two times higher (HR 2.2; 95% CI, 1.3–3.7) in patients with elevated GDF15 plasma levels (>116 pM per 5 *μ*l), which can be compared with the results documented on patients with elevated GDF15 serum levels (>1150 pg ml^−1^) by Brown *et al* (2003) (OR 2.11; 95% CI, 1.04–4.28). In our study, the plasma levels of GDF15 were not significantly different between patients with or without recurrence in stages I–III, even though there was a trend of a higher plasma levels in patients with recurrence in stage III.

The presence of vascular invasion is known to be an independent prognostic factor for both colon (Shepherd *et al*, 1989; Petersen *et al*, 2002) and rectal cancer (Talbot *et al*, 1980; Willett *et al*, 1999; Smith *et al*, 2008). A study by Petersen *et al* (2002) even proposed that the presence of vascular invasion along with three other pathologically determined parameters could be used to make decisions regarding adjuvant therapy in stage II CRC. In our study, increased GDF15 expression was negatively associated with vascular invasion. This observation is supported by a previous report on the anti-angiogenic activity of GDF15 (Ferrari *et al*, 2005) in which GDF15 was demonstrated both *in vivo* and *in vitro* to inhibit angiogenesis in endothelial cells. This inconsistent finding, of decreased vascular invasion and higher risk for recurrences, could be a result of the increased likelihood of a significant outcome by chance because of multiple testing; nevertheless, it could also be explained by the divergent molecular mechanisms of GDF15. GDF15 has been implicated both as a promoter and inhibitor of tumour growth (Tan *et al*, 2000; Baek *et al*, 2001; Levy and Hill, 2006; Abd El-Aziz *et al*, 2007; Johnen *et al*, 2007). The conflicting results between *in vitro* and *in vivo* studies regarding the role of GDF15 in tumourigenesis can probably be attributed to the interaction of the tumour with the microenvironment (Albertoni *et al*, 2002; Krieg *et al*, 2010). The current belief is that GDF15 has pleiotropic effects in cancer progression by functioning as a tumour suppressor inhibiting tumour growth, inducing apoptosis in early stages, although it promotes proliferation, migration, invasion and metastasis in more advanced disease stages (Mimeault and Batra, 2010). This belief of a dual and stage-dependent role of GDF15 in tumourigenesis could potentially, in our study, explain why a high intensity of GDF15 expression was associated with a shorter time to recurrence in stage III but not in stage II disease.

We confirmed a difference in the intensity of immunoreactivity of GDF15 between normal mucosa and invasive tumour tissue, but failed to demonstrate a difference in the fraction of GDF15-positive cells between normal tissue and invasive tumour tissue. This indicates that there might be a pathophysiological distinction between activity of GDF15 expression (measured as intensity) and actual number of cells expressing GDF15 (measured as fraction) between invasive tumour tissue and normal mucosa, supporting that intensity of immunoreactivity rather than fraction of positive cells for GDF15 better serves as a prognostic marker in CRC.

Plasma levels of GDF15 have been studied as a biomarker in cardiovascular disease (Hochholzer *et al*, 2010) and elevated levels have been seen in metastatic CRC, breast and prostate carcinomas compared with normal controls (Welsh *et al*, 2003). The correlation between GDF15 plasma levels and CEA in the whole cohort including all disease stages was weak, but when analysing patients with stage IV disease, a stronger correlation was observed. We demonstrated a gradual trend of increasing GDF15 plasma levels from stage I to IV, whereas the CEA levels were low in patients with early disease stages, but significantly elevated in stage IV patients. The predictive value of CEA for detecting colorectal cancer in early stages is known to be low (Fletcher, 1986), although there is clear evidence that preoperative plasma CEA levels correlate with stage and serve as an independent prognostic factor of survival (Wanebo *et al*, 1978; Wolmark *et al*, 1984; Slentz *et al*, 1994). Consistent with a previous study by Brown *et al* (2003), GDF15 plasma levels in our study was an independent prognostic factor of survival supporting that measurement of GDF15 levels in plasma might add additional prognostic information in patients with CRC. The samples in our study were strategically selected to get a more reliable estimate the GDF15 plasma analyses but still limited by the small sample size and, therefore, decreased the power and the precision of the results.

Other issues regarding immunohistochemistry related to different fixation techniques and duration of fixation of the tumour tissue could potentially also have influence our results (Atkins *et al*, 2004; Leong, 2004; Paavilainen *et al*, 2010). However, in our study all tissue specimens were handled at the same pathology department. Consequently, in this prospectively collected material, we predict low variability of the actual handling of the tissue specimens. The antibody used in the immunohistochemistry staining had a high specificity on protein array and a single band corresponding to the predicted size in kDa on the western blot. Therefore, we believe that it is less likely that the specificity of the antibody influenced our results.

In conclusion, we have demonstrated that increased GDF15 expression may have a negative prognostic value in patients curatively operated for CRC stages I–III and III disease. However, the actual role of GDF15 in tumourigenesis is still unclear, and further research regarding both its pathophysiological role and clinical use as a prognostic marker for CRC is needed preferably in prospective clinical trials.

## Figures and Tables

**Table 1:** Expression of GDF15 in normal tissue adjacent to an invasive tumour (a) and invasive tumours (b)

	**GDF15 intensity of immunoreactivity staining**				
**GDF15 fraction of GDF15 stained positive cells**	**0**	**1**	**2**	**3**	**Total**
*(a) Normal tissue*					
0	70	10	14	3	97
1	0	1	2	4	7
2	0	4	11	1	16
3	0	2	8	0	10
Total	70	17	35	8	130
					
*(b) Tumour tissue*					
0	29	18	81	35	163
1	0	4	45	54	103
2	0	0	31	17	48
3	0	0	3	0	3
Total	29	22	160	106	317

Abbreviation: GDF15=growth differentiation factor 15.

The intensity of immunoreactivity for GDF15 (negative (0), weak (1), moderate (2), or strong (3)) and fraction of positive cells (<1% (0), 1–25% (1)), 25–75% (2), or >75% (3)) are presented in the tables.

**Table 2:**
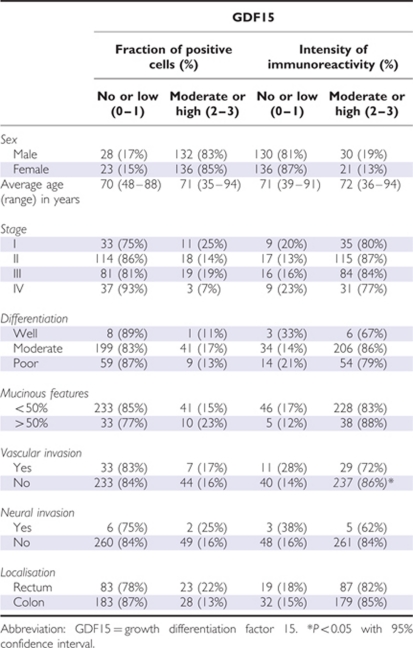
Clinical and pathological characteristics in association with the distribution of fraction of positive cells and intensity of immunoreactivity for GDF15 analysed by immunohistochemistry in 320 patients with colorectal cancer

**Table 3:**
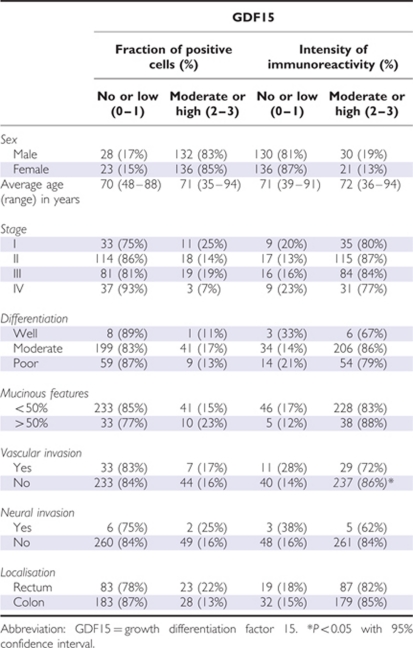
Results from the univariate and multivariate Cox regression analyses estimating the effect of the intensity, fraction of GDF15 expression on time to recurrence in patients curatively operated for CRC in stages I–III (*n*=277) and in stage II (*n*=131) and stage III (*n*=100) separately

**Table 4:** Median plasma levels of GDF15 in patients with stages I–IV with and without recurrence

**Median plasma levels of GDF15 (range)**														
**Stage**	**I**	***N***	**II**	***N***	**III**	***N***	**IV**	***N***	**II–III**	***N***	**I–III**	***N***	**I–IV**	***N***
No recurrence	94.0 (23–318)	8	91.5 (68–675)	11	115.8 (23–516)	9		n/a	104.5 (23–675)	20	95.8 (23–675)	28	95.8 (23–675)	28
Recurrence	351.2	1	84.1 (43–235)	10	205.9 (51–855)	10	146.5 (39–1008)	8	144.7 (43–855)	20	144.7 (43–855)	21	144.7 (38–1008)	29
*P*-value	0.121	9	0.091	21	0.072	19	n/a	8	0.93	40	0.6	49	0.49	57

Abbreviations: n/a=not applicable; GDF15=Growth differentiation factor 15.

The concentrations of GDF15 are in pM in 5 μl of undiluted plasma.

## References

[bib1] Abd El-Aziz SH, Endo Y, Miyamaori H, Takino T, Sato H (2007) Cleavage of growth differentiation factor 15 (GDF15) by membrane type 1-matrix metalloproteinase abrogates GDF15-mediated suppression of tumor cell growth. Cancer Sci 98(9): 1330–1335 17640303 10.1111/j.1349-7006.2007.00547.x PMC11158783

[bib2] Albertoni M, Shaw PH, Nozaki M, Godard S, Tenan M, Hamou MF, Fairlie DW, Breit SN, Paralkar VM, de Tribolet N, Van Meir EG, Hegi ME (2002) Anoxia induces macrophage inhibitory cytokine-1 (MIC-1) in glioblastoma cells independently of p53 and HIF-1. Oncogene 21(27): 4212–4219 12082608 10.1038/sj.onc.1205610

[bib3] Amado RG, Wolf M, Peeters M, Van Cutsem E, Siena S, Freeman DJ, Juan T, Sikorski R, Suggs S, Radinsky R, Patterson SD, Chang DD (2008) Wild-type KRAS is required for panitumumab efficacy in patients with metastatic colorectal cancer. J Clin Oncol 26(10): 1626–1634 18316791 10.1200/JCO.2007.14.7116

[bib4] Andre T, Boni C, Mounedji-Boudiaf L, Navarro M, Tabernero J, Hickish T, Topham C, Zaninelli M, Clingan P, Bridgewater J, Tabah-Fisch I, de Gramont A (2004) Oxaliplatin, fluorouracil, and leucovorin as adjuvant treatment for colon cancer. N Engl J Med 350(23): 2343–2351 15175436 10.1056/NEJMoa032709

[bib5] Atkins D, Reiffen KA, Tegtmeier CL, Winther H, Bonato MS, Storkel S (2004) Immunohistochemical detection of EGFR in paraffin-embedded tumor tissues: variation in staining intensity due to choice of fixative and storage time of tissue sections. J Histochem Cytochem 52(7): 893–901 15208356 10.1369/jhc.3A6195.2004

[bib6] Baek SJ, Kim KS, Nixon JB, Wilson LC, Eling TE (2001) Cyclooxygenase inhibitors regulate the expression of a TGF-beta superfamily member that has proapoptotic and antitumorigenic activities. Mol Pharmacol 59(4): 901–908 11259636

[bib7] Brown DA, Stephan C, Ward RL, Law M, Hunter M, Bauskin AR, Amin J, Jung K, Diamandis EP, Hampton GM, Russell PJ, Giles GG, Breit SN (2006) Measurement of serum levels of macrophage inhibitory cytokine 1 combined with prostate-specific antigen improves prostate cancer diagnosis. Clin Cancer Res 12(1): 89–96 16397029 10.1158/1078-0432.CCR-05-1331

[bib8] Brown DA, Ward RL, Buckhaults P, Liu T, Romans KE, Hawkins NJ, Bauskin AR, Kinzler KW, Vogelstein B, Breit SN (2003) MIC-1 serum level and genotype: associations with progress and prognosis of colorectal carcinoma. Clin Cancer Res 9(7): 2642–2650 12855642

[bib9] Darmanis S, Nong RY, Hammond M, Gu J, Alderborn A, Vanelid J, Siegbahn A, Gustafsdottir S, Ericsson O, Landegren U, Kamali-Moghaddam M (2010) Sensitive plasma protein analysis by microparticle-based proximity ligation assays. Mol Cell Proteomics 9(2): 327–335 19955079 10.1074/mcp.M900248-MCP200 PMC2830843

[bib10] de Wit NJ, Rijntjes J, Diepstra JH, van Kuppevelt TH, Weidle UH, Ruiter DJ, van Muijen GN (2005) Analysis of differential gene expression in human melanocytic tumour lesions by custom made oligonucleotide arrays. Br J Cancer 92(12): 2249–2261 15900300 10.1038/sj.bjc.6602612 PMC2361822

[bib11] Elsaleh H, Shannon B, Iacopetta B (2001) Microsatellite instability as a molecular marker for very good survival in colorectal cancer patients receiving adjuvant chemotherapy. Gastroenterology 120(5): 1309–1310 11288748 10.1053/gast.2001.23646

[bib12] Fairlie WD, Moore AG, Bauskin AR, Russell PK, Zhang HP, Breit SN (1999) MIC-1 is a novel TGF-beta superfamily cytokine associated with macrophage activation. J Leukoc Biol 65(1): 2–5 9886240 10.1002/jlb.65.1.2

[bib13] Ferrari N, Pfeffer U, Dell’Eva R, Ambrosini C, Noonan DM, Albini A (2005) The transforming growth factor-beta family members bone morphogenetic protein-2 and macrophage inhibitory cytokine-1 as mediators of the antiangiogenic activity of N-(4-hydroxyphenyl)retinamide. Clin Cancer Res 11(12): 4610–4619 15958647 10.1158/1078-0432.CCR-04-2210

[bib14] Fletcher RH (1986) Carcinoembryonic antigen. Ann Intern Med 104(1): 66–73 3510056 10.7326/0003-4819-104-1-66

[bib15] Gill S, Loprinzi CL, Sargent DJ, Thome SD, Alberts SR, Haller DG, Benedetti J, Francini G, Shepherd LE, Francois Seitz J, Labianca R, Chen W, Cha SS, Heldebrant MP, Goldberg RM (2004) Pooled analysis of fluorouracil-based adjuvant therapy for stage II and III colon cancer: who benefits and by how much? J Clin Oncol 22(10): 1797–1806 15067028 10.1200/JCO.2004.09.059

[bib16] Hemminki A, Mecklin JP, Jarvinen H, Aaltonen LA, Joensuu H (2000) Microsatellite instability is a favourable prognostic indicator in patients with colorectal cancer receiving chemotherapy. Gastroenterology 119(4): 921–928 11040179 10.1053/gast.2000.18161

[bib17] Hochholzer W, Morrow DA, Giugliano RP (2010) Novel biomarkers in cardiovascular disease: update 2010. Am Heart J 160(4): 583–594 20934551 10.1016/j.ahj.2010.06.010

[bib18] Johnen H, Lin S, Kuffner T, Brown DA, Tsai VW, Bauskin AR, Wu L, Pankhurst G, Jiang L, Junankar S, Hunter M, Fairlie WD, Lee NJ, Enriquez RF, Baldock PA, Corey E, Apple FS, Murakami MM, Lin EJ, Wang C, During MJ, Sainsbury A, Herzog H, Breit SN (2007) Tumor-induced anorexia and weight loss are mediated by the TGF-beta superfamily cytokine MIC-1. Nat Med 13(11): 1333–1340 17982462 10.1038/nm1677

[bib19] Jover R, Zapater P, Castells A, Llor X, Andreu M, Cubiella J, Pinol V, Xicola RM, Bujanda L, Rene JM, Clofent J, Bessa X, Morillas JD, Nicolas-Perez D, Paya A, Alenda C (2006) Mismatch repair status in the prediction of benefit from adjuvant fluorouracil chemotherapy in colorectal cancer. Gut 55(6): 848–855 16299036 10.1136/gut.2005.073015 PMC1856227

[bib20] Karapetis CS, Khambata-Ford S, Jonker DJ, O’Callaghan CJ, Tu D, Tebbutt NC, Simes RJ, Chalchal H, Shapiro JD, Robitaille S, Price TJ, Shepherd L, Au HJ, Langer C, Moore MJ, Zalcberg JR (2008) K-ras mutations and benefit from cetuximab in advanced colorectal cancer. N Engl J Med 359(17): 1757–1765 18946061 10.1056/NEJMoa0804385

[bib21] Koopmann J, Buckhaults P, Brown DA, Zahurak ML, Sato N, Fukushima N, Sokoll LJ, Chan DW, Yeo CJ, Hruban RH, Breit SN, Kinzler KW, Vogelstein B, Goggins M (2004) Serum macrophage inhibitory cytokine 1 as a marker of pancreatic and other periampullary cancers. Clin Cancer Res 10(7): 2386–2392 15073115 10.1158/1078-0432.ccr-03-0165

[bib22] Krieg AJ, Rankin EB, Chan D, Razorenova O, Fernandez S, Giaccia AJ (2010) Regulation of the histone demethylase JMJD1A by hypoxia-inducible factor 1 alpha enhances hypoxic gene expression and tumor growth. Mol Cell Biol 30(1): 344–353 19858293 10.1128/MCB.00444-09 PMC2798291

[bib23] Leong AS (2004) Pitfalls in diagnostic immunohistology. Adv Anat Pathol 11(2): 86–93 15090844 10.1097/00125480-200403000-00002

[bib24] Levy L, Hill CS (2006) Alterations in components of the TGF-beta superfamily signaling pathways in human cancer. Cytokine Growth Factor Rev 17(1–2): 41–58 16310402 10.1016/j.cytogfr.2005.09.009

[bib25] Mimeault M, Batra SK (2010) Divergent molecular mechanisms underlying the pleiotropic functions of macrophage inhibitory cytokine-1 in cancer. J Cell Physiol 224(3): 626–635 20578239 10.1002/jcp.22196 PMC2932466

[bib26] Ohrling K, Edler D, Hallstrom M, Ragnhammar P (2010) Mismatch repair protein expression is an independent prognostic factor in sporadic colorectal cancer. Acta Oncol 49(6): 797–804 20307245 10.3109/02841861003705786

[bib27] Paavilainen L, Edvinsson A, Asplund A, Hober S, Kampf C, Ponten F, Wester K (2010) The impact of tissue fixatives on morphology and antibody-based protein profiling in tissues and cells. J Histochem Cytochem 58(3): 237–246 19901271 10.1369/jhc.2009.954321 PMC2825489

[bib28] Parkin DM, Bray F, Ferlay J, Pisani P (2005) Global cancer statistics, 2002. CA Cancer J Clin 55(2): 74–108 15761078 10.3322/canjclin.55.2.74

[bib29] Petersen VC, Baxter KJ, Love SB, Shepherd NA (2002) Identification of objective pathological prognostic determinants and models of prognosis in Dukes’ B colon cancer. Gut 51(1): 65–69 12077094 10.1136/gut.51.1.65 PMC1773289

[bib30] Ponten F, Jirstrom K, Uhlen M (2008) The Human Protein Atlas--a tool for pathology. J Pathol 216(4): 387–393 18853439 10.1002/path.2440

[bib31] Popat S, Hubner R, Houlston RS (2005). Systematic review of microsatellite instability and colorectal cancer prognosis. J Clin Oncol 23: 609–618 15659508 10.1200/JCO.2005.01.086

[bib32] Punt CJ, Buyse M, Kohne CH, Hohenberger P, Labianca R, Schmoll HJ, Pahlman L, Sobrero A, Douillard JY (2007) Endpoints in adjuvant treatment trials: a systematic review of the literature in colon cancer and proposed definitions for future trials. J Natl Cancer Inst 99(13): 998–1003 17596575 10.1093/jnci/djm024

[bib33] Gray R, Barnwell J, McConkey C, Hills RK, Williams NS, Kerr DJ (2007) Adjuvant chemotherapy versus observation in patients with colorectal cancer: a randomised study. Lancet 370(9604): 2020–2029 18083404 10.1016/S0140-6736(07)61866-2

[bib34] Ragnhammar P, Hafstrom L, Nygren P, Glimelius B (2001) A systematic overview of chemotherapy effects in colorectal cancer. Acta Oncol 40(2–3): 282–308 11441937 10.1080/02841860151116367

[bib35] Seetoo DQ, Crowe PJ, Russell PJ, Yang JL (2003) Quantitative expression of protein markers of plasminogen activation system in prognosis of colorectal cancer. J Surg Oncol 82(3): 184–193 12619063 10.1002/jso.10210

[bib36] Shepherd NA, Saraga EP, Love SB, Jass JR (1989) Prognostic factors in colonic cancer. Histopathology 14(6): 613–620 2759558 10.1111/j.1365-2559.1989.tb02202.x

[bib37] Slentz K, Senagore A, Hibbert J, Mazier WP, Talbott TM (1994) Can preoperative and postoperative CEA predict survival after colon cancer resection? Am Surg 60(7): 528–531; discussion 531–532 8010568

[bib38] Smith NJ, Shihab O, Arnaout A, Swift RI, Brown G (2008) MRI for detection of extramural vascular invasion in rectal cancer. AJR Am J Roentgenol 191(5): 1517–1522 18941094 10.2214/AJR.08.1298

[bib39] Talbot IC, Ritchie S, Leighton MH, Hughes AO, Bussey HJ, Morson BC (1980) The clinical significance of invasion of veins by rectal cancer. Br J Surg 67(6): 439–442 7388345 10.1002/bjs.1800670619

[bib40] Tan M, Wang Y, Guan K, Sun Y (2000) PTGF-beta, a type beta transforming growth factor (TGF-beta) superfamily member, is a p53 target gene that inhibits tumor cell growth via TGF-beta signaling pathway. Proc Natl Acad Sci USA 97(1): 109–114 10618379 10.1073/pnas.97.1.109 PMC26624

[bib41] Uhlen M, Bjorling E, Agaton C, Szigyarto CA, Amini B, Andersen E, Andersson AC, Angelidou P, Asplund A, Asplund C, Berglund L, Bergstrom K, Brumer H, Cerjan D, Ekstrom M, Elobeid A, Eriksson C, Fagerberg L, Falk R, Fall J, Forsberg M, Bjorklund MG, Gumbel K, Halimi A, Hallin I, Hamsten C, Hansson M, Hedhammar M, Hercules G, Kampf C, Larsson K, Lindskog M, Lodewyckx W, Lund J, Lundeberg J, Magnusson K, Malm E, Nilsson P, Odling J, Oksvold P, Olsson I, Oster E, Ottosson J, Paavilainen L, Persson A, Rimini R, Rockberg J, Runeson M, Sivertsson A, Skollermo A, Steen J, Stenvall M, Sterky F, Stromberg S, Sundberg M, Tegel H, Tourle S, Wahlund E, Walden A, Wan J, Wernerus H, Westberg J, Wester K, Wrethagen U, Xu LL, Hober S, Ponten F (2005) A human protein atlas for normal and cancer tissues based on antibody proteomics. Mol Cell Proteomics 4(12): 1920–1932 16127175 10.1074/mcp.M500279-MCP200

[bib42] Wanebo HJ, Rao B, Pinsky CM, Hoffman RG, Stearns M, Schwartz MK, Oettgen HF (1978) Preoperative carcinoembryonic antigen level as a prognostic indicator in colorectal cancer. N Engl J Med 299(9): 448–451 683276 10.1056/NEJM197808312990904

[bib43] Watanabe T, Wu TT, Catalano PJ, Ueki T, Satriano R, Haller DG, Benson III AB, Hamilton SR (2001) Molecular predictors of survival after adjuvant chemotherapy for colon cancer. N Engl J Med 344(16): 1196–1206 11309634 10.1056/NEJM200104193441603 PMC3584633

[bib44] Weber F, Shen L, Aldred MA, Morrison CD, Frilling A, Saji M, Schuppert F, Broelsch CE, Ringel MD, Eng C (2005) Genetic classification of benign and malignant thyroid follicular neoplasia based on a three-gene combination. J Clin Endocrinol Metab 90(5): 2512–2521 15713710 10.1210/jc.2004-2028

[bib45] Welsh JB, Sapinoso LM, Kern SG, Brown DA, Liu T, Bauskin AR, Ward RL, Hawkins NJ, Quinn DI, Russell PJ, Sutherland RL, Breit SN, Moskaluk CA, Frierson Jr HF, Hampton GM (2003) Large-scale delineation of secreted protein biomarkers overexpressed in cancer tissue and serum. Proc Natl Acad Sci USA 100(6): 3410–3415 12624183 10.1073/pnas.0530278100 PMC152306

[bib46] Willett CG, Badizadegan K, Ancukiewicz M, Shellito PC (1999) Prognostic factors in stage T3N0 rectal cancer: do all patients require postoperative pelvic irradiation and chemotherapy? Dis Colon Rectum 42(2): 167–173 10211491 10.1007/BF02237122

[bib47] Wolmark N, Fisher B, Wieand HS, Henry RS, Lerner H, Legault-Poisson S, Deckers PJ, Dimitrov N, Gordon PH, Jochimsen P (1984) The prognostic significance of preoperative carcinoembryonic antigen levels in colorectal cancer. Results from NSABP (National Surgical Adjuvant Breast and Bowel Project) clinical trials. Ann Surg 199(4): 375–382 6370155 10.1097/00000658-198404000-00001 PMC1353353

[bib48] Xue H, Lu B, Zhang J, Wu M, Huang Q, Wu Q, Sheng H, Wu D, Hu J, Lai M (2010) Identification of serum biomarkers for colorectal cancer metastasis using a differential secretome approach. J Proteome Res 9(1): 545–555 19924834 10.1021/pr9008817

